# Therapeutic Efficacy of Artemether-Lumefantrine (Coartem®) for the Treatment of Uncomplicated Falciparum Malaria in Africa: A Systematic Review

**DOI:** 10.1155/2020/7371681

**Published:** 2020-10-20

**Authors:** Awoke Derbie, Daniel Mekonnen, Meseret Adugna, Biruk Yeshitela, Yimtubezinash Woldeamanuel, Tamrat Abebe

**Affiliations:** ^1^Department of Medical Microbiology, College of Medicine and Health Sciences, Bahir Dar University, Ethiopia; ^2^Centre for Innovative Drug Development and Therapeutic Trials for Africa (CDT-Africa), Addis Ababa University, Ethiopia; ^3^Department of Health Biotechnology, Biotechnology Research Institute, Bahir Dar University, Ethiopia; ^4^Department of Internal Medicine, College of Medicine and Health Sciences, Bahir Dar University, Ethiopia; ^5^Armaur Hanssen Research Institute (AHRI), Addis Ababa, Ethiopia; ^6^Department of Medical Microbiology, Immunology and Parasitology, School of Medicine, College of Health Sciences, Addis Ababa University, Ethiopia

## Abstract

**Background:**

Africa still bears the largest burden of malaria as the majority of infections in the continent are caused by *P. falciparum*. Artemether-lumefantrine (AL, Coartem®) is the most widely used artemisinin-based combination therapy (ACT), for treating uncomplicated falciparum malaria globally. However, the development of resistance to antimalarial drugs is a major challenge for malaria control. In this review, the efficacy of AL for the treatment of uncomplicated falciparum malaria in Africa was evaluated.

**Methods:**

Articles published between January 2015 and July 2019 were systematically searched using comprehensive search strings from PubMed/Medline, SCOPUS, and grey literature from Google Scholar. Interventional studies that followed patients for at least 28 days were included. Two reviewers independently assessed study eligibility, extracted data, and assessed risk of bias. All the included articles were measured to be good quality. While computing the efficacy of AL, polymerase chain reaction (PCR)–corrected cure rate (adequate clinical and parasitological response, ACPR) at day 28 was considered as the main endpoint. Meta-analysis was computed using STATA v 15 to calculate the pooled ACPR.

**Results:**

In this review, 39 articles that reported the treatment outcome of 8,320 patients were included. After 28 days of follow-up, the pooled PCR uncorrected and corrected APCR was at 87% (95% CI: 85-90%) and 97.0% (95% CI: 96-98%), respectively. Moreover, the proportion of early treatment failure (ETF) was almost 0%, while most of the included articles reported <8% late treatment failures. The reinfection and recrudescence rate was less than 10% and 2.6%, respectively, within 28 days. We noted rapid fever and parasite clearance in which greater than 93% and 94% patients were parasite and fever free at day three following AL treatment.

**Conclusions:**

This review discovered that despite more than a decade since its introduction, Coartem® remains effective and thus could continue to be the drug of choice for the treatment of uncomplicated falciparum malaria for all age groups in Africa. However, the risk of new emerging resistance for this combination warrants regular monitoring of its efficacy across the continent.

## 1. Background

Malaria is one of the most severe public health problems and a leading cause of death in many developing countries, where young children and pregnant women are the groups most affected [1]. Although malaria has taken a staggering toll on human health in the past, the 21^st^ century seems poised to consider its elimination and eradication [2, 3]. There are five parasite species that cause malaria in humans, and two of these species; *P. falciparum* and *P. vivax* pose the greatest threat globally [4, 5]. *P. falciparum* is the most prevalent malaria parasite in Africa [6]. Although the burden of falciparum malaria is gradually declining in many parts of the continent, it is characterized by spatial and temporal variability that presents new and evolving challenges for malaria control programs [5]. The long lifespan and strong human-biting habit of the African vector species, mainly *Anopheles gambiae*, *A. funestus*, and *A. arabiensis*, is the main reason why more than 90% of the world's malaria cases are in Africa [4].

A patient who presents with symptoms of malaria and a positive parasitological test (microscopy or Rapid diagnostic test (RDT)) but with no features of severe malaria is defined as having uncomplicated malaria [3]. Uncomplicated falciparum malaria can progress rapidly to severe forms of the disease (within hours), especially in people with no or low immunity, and severe falciparum malaria is almost always fatal without treatment [3].

While the exact numbers may be uncertain and under reporting is inevitable, according to the latest World Health Organization (WHO) malaria report, an estimated 219 million cases of malaria occurred worldwide in 2017. In same year, the African region was home to 92% of malaria cases and 93% of malaria deaths. Fifteen countries in sub-Saharan Africa and India carried almost 80% of the global malaria burden. Five countries accounted for nearly half of all malaria cases worldwide: Nigeria (25%), Democratic Republic of the Congo (11%), Mozambique (5%), India (4%), and Uganda (4%) [6]. Similarly, in 2017, there were an estimated 435,000 deaths from malaria globally. In same year, children <5 years accounted for 61% (266,000) of all malaria deaths globally [6].

Malaria case management consists of early diagnosis and prompt treatment [3]. In recent years with increases in diagnostic testing, artemisinin-based combination therapy (ACT) is becoming more targeted towards patients who tested positive for malaria [6]. Clinical failure and spread of resistance to chloroquine and sulfadoxine-pyrimethamine led to the introduction of ACTs in Africa [2]. ACTs are combinations of an artemisinin derivative and another structurally unrelated and more slowly eliminated antimalarial [7] that are recommended by WHO and are now generally accepted as the best option for the treatment of uncomplicated *falciparum* malaria [3]. They are rapidly and reliably effective. Efficacy is determined by the drug partnering the artemisinin derivative and for artesunate–mefloquine, artemether–lumefantrine (AL), and dihydroartemisinin–piperaquine; this usually exceeds 95% [7]. The choice of appropriate ACT depends on factors like cost, efficacy, safety, reinfection rate, and simplicity of administration [8]. In Africa, AL is the most widely used [9] ACT, whereas artesunate–mefloquine is used infrequently because of a perceived poor tolerance to mefloquine [10].

AL or Coartem® (Novartis Pharma AG, Basel Switzerland) was the first fixed dose combination of an artemisinin derivative with a second unrelated antimalarial compound. It is a safe and effective treatment for children and adults with *P. falciparum* malaria. Both components are *blood schizontocides*. The dual mechanisms of action of AL provide rapid and sustained parasite clearance [11]. Lumefantrine (formerly benflumetol) is an aryl amino-alcohol in the same general group as mefloquine and halofantrine [7].

Resistance to antimalarial medicines is a great public health challenge. Antimalarial drug resistance may continue to be a leading threat to ongoing malaria control efforts and calls for continued monitoring of the efficacy of these drugs for drug policy input [12]. Regular efficacy monitoring of ACT shall be conducted every 2-3 years [13]. Artemisinin resistance, defined as delayed parasite clearance, has emerged recently in southeastern Asia [14–16] which is of the highest concern [17]. While there are so far no multiple reports on artemisinin resistance in Africa and South America, a declining parasitological response to AL was noticed in Nigeria over the last 10 years [18]. On top of this, the emergence and spread of artemisinin resistance worldwide is a present danger and needs more attention [16, 19]. Hence, regular surveillance and monitoring measures are recommended by WHO to help early detection of drug-resistant strains of plasmodium and contain their rapid spread [3, 19, 20].

As there is quite limited reviewed data on the topic of AL resistance in Africa, there is a need to better understand the dynamics of parasite clearance in patients treated with ACT in order to better detect the emergence of AL resistance for intervention [21]. It is also stated that the effectiveness of artemisinin derivatives in Africa must be monitored to detect resistance early [22]. Hence, data is required on this field to inform policy makers. Therefore, this systematic review was conducted aimed at determining the therapeutic efficacy of AL in the treatment of uncomplicated falciparum in Africa; the data will serve as an input to evaluate the current malaria treatment policy in the continent.

### 1.1. Review Question

This systematic review stands with the following question: what is the therapeutic efficacy of AL for the treatment of uncomplicated falciparum malaria in Africa over the last five years?

### 1.2. Objective

The main aim of the review was to summarize the latest five years data on the therapeutic efficacy of AL for the treatment of uncomplicated falciparum malaria in the African context.

## 2. Methods

### 2.1. Protocol Registration

In accordance with the Preferred Reporting Items for Systematic Reviews and Meta-Analysis (PRISMA) guideline, the review protocol was registered on the International Prospective Register of Systematic Reviews (PROSPERO) with a registration number CRD42020142590.

### 2.2. Eligibility Criteria

Studies were selected based on the following criterion. *Study design*: interventional studies that reported the therapeutic efficacy of AL for the treatment of uncomplicated malaria. *Participants*: *P. falciparum*-infected patients irrespective of gender and age group. *Interventions*: a standard six-dose regimen of AL over three days followed up for 28 days to measure its therapeutic responses. *Setting*: we included studies with the outcome of interest reported in Africa. *Language and publication*: we included peer-reviewed journal articles and unpublished findings reported in the English language in the last five years (January 2015 to July 2019).

### 2.3. Information Sources and Search Strategy

This review was done following PRISMA [23]. A computerized systematic strategy was adopted to search papers in PubMed/Medline and SCOPUS, the last search was conducted on July 15, 2019. Manual search from Google scholar and Google databases was also done for grey literature. The search terms were developed in line with the Medical Subject Headings (MeSH) thesaurus using a combination of the big ideas (or “key terms”) which derived from the research question. The reference lists of retrieved articles were probed (forward and backward searching) to identify articles that were not retrieved from databases and our manual search. The first two authors, AD and DM, searched the articles independently.

The domains of the search terms were: “efficacy”, “therapeutic efficacy”, “artemether-lumefantrine”, ”Coartem”, “*Plasmodium falciparum* malaria”, “faciparum malaria”, “antimalarial drug”, and “Africa”. We combined these terms using the Boolean operator “OR” and “AND” accordingly. Full search strategy for the two databases is attached separately as a supplement.

### 2.4. Study Selection

Studies that have been published in the last five years (2015 to 2019) and reported the therapeutic efficacy of AL for the treatment of uncomplicated falciparum malaria in African context were included. Searched articles were directly imported and handled using EndNote X5 citation manager (Thomson Reuters, New York, USA). Based on the PRISMA protocol, duplicated articles were excluded, and the titles and abstracts of the remaining papers were screened independently for inclusion in full text evaluation by the first two authors. Differences between the reviewers were resolved through discussion.

### 2.5. Data Collection Process and Data Items

The Joanna Briggs Institute (JBI) data extraction tool was adopted for data extraction. Relevant data such as the name of the first author, year of publication, country where the study was conducted, mean/median age of the study participants, proportion of male participants, type of the study design, total number of the study participants, follow-up period, baseline characteristics of study participants (mean body weight, body temperature, hemoglobin, geometric mean parasite density, and proportion of gametocytes), and fever and parasite clearance rates were extracted from the included articles. Moreover, based on the WHO recommendation [24], the treatment failure (early treatment failure (ETF), late parasitological failure (LPF), late clinical failure (LCF)), and the cure rate in terms of adequate clinical and parasitological response (ACPR) were extracted from each study.

### 2.6. Methodological Quality Appraisal of the Included Studies

Validity and methodological quality of all included studies were assessed using the national institute of health (NIH) study quality assessment tool for intervention studies [25]. The tool consists of fourteen criteria that were checked as “yes,” “no,” or “not applicable/cannot determined or not reported.” The tool asks about treatment allocation, randomization and blinding, inclusion and exclusion criteria, the sample size, lost to follow-up, and the exposure and outcome measurement of each included studies. After carefully evaluating the included articles against each criterion, studies were finally classified into three groups; a study that fulfilled >80% of the criteria was considered as “good quality.” Similarly, a study that scored 50-80% and <50% were rated as “fair” and “poor” quality, respectively.

### 2.7. Data Synthesis

The data extracted from the included studies were fed into Microsoft Excel. Descriptive statistics, such as simple counts, ranges, and percentages, were used to present the synthesized data. A systematic narrative synthesis was provided in which summary results were presented using text and table. To compute the pooled ACPR with its 95% CI, meta-analysis was done using STATA v15 (Stata Corp. College Station, TX, USA) assuming a random effect model. In this review, following AL therapy, the primary endpoint (or efficacy evaluation) was cure rate (or ACPR), corrected to exclude reinfection using polymerase-chain reaction (PCR), at day 28.

### 2.8. Operational Definition

The definition of the following terms was adopted from cited references [3, 26–31].

Adequate clinical and parasitological response (ACPR): refers to *P. falciparum* parasitological clearance at day 28 irrespective of axillary, oral, rectal, or tympanic temperature without previously meeting the criteria of early treatment failure or PCR corrected late treatment failure.

Early treatment failure (ETF): signs of severe malaria/clinical deterioration requiring rescue medication on days 0, 1, 2, or 3, in the presence of *P. falciparum* parasitemia.

Late clinical failure (LCF): signs of severe malaria/ clinical deterioration requiring rescue medication after day 3 in the presence of *P. falciparum* parasitemia, without previously meeting any of the criteria of ETF.

Late parasitological failure (LPF): presence of *P. falciparum* parasitemia on any day from day 7 onward and the absence of fever without previously meeting any of the criteria of ETF/or LCF.

PCR-corrected: refers to the use of molecular testing to differentiate recrudescence from reinfection when evaluating efficacy. Recurrent parasitemia classified as recrudescence if it was due to the same parasite strain as that on day 0 (if similar alleles were found in the pre- and posttreatment samples) and as a new infection if it was due to a genetically different strain (if the alleles of the pre- and posttreatment samples were distinct).

## 3. Results

### 3.1. Search Results

**Figure 1:**
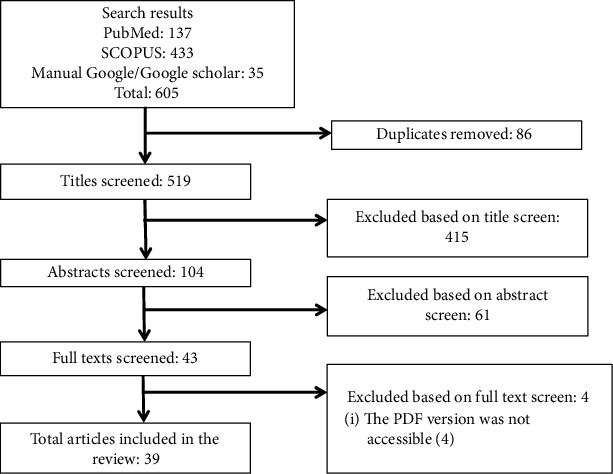
The PRISMA flow diagram of literature selection.

From the systematically searched databases and other sources, a total of 605 articles were retrieved and sequentially screened for final inclusion. As depicted in Figure 1, screening was based on the PRISMA flow chart which was adapted from the PRISMA guideline [23]. After removing the duplicate, 519 were screened by title; then, 415 were removed. Consequently, 61 were excluded by abstract and 4 by full text with justifiable reasons. Lastly, a total of 39 studies met our inclusion criteria and included in this review for analysis.

### 3.2. Characteristics of the Included Studies

The characteristic of the included articles is summarized in (Table 1). The studies were reported from 20 different African countries, representing the five regions of the continent (north, south, east, west, and central Africa). The articles reported the efficacy of AL for the treatment of uncomplicated falciparum malaria in either randomized clinical trials or observational single arm cohort studies. The WHO guide for surveillance of antimalarial drug efficacy was used by the studies to select study participants and to conduct the study.

The number of participants in each included study varied from 33 to 595. Majority of the included articles 31 (79.5%) measured the efficacy of AL in terms of ACPR at day 28, while in the remaining studies at 7 (17.9%) and 1 (2.7%), the follow-up period was 42 and 63 days, respectively. In total, the review contains reports of 8,320 patients. Most 31 (79.5%) of the studies included patients who were older than six months, while in the remaining studies at 7 (17.9%) included all age groups and 1 (2.6%) include only pregnant women.

### 3.3. Methodological Quality of Included Studies

The methodological quality of all the included studies was assessed using the national institute of health (NIH) study quality assessment tool for intervention studies [25]. Providentially all the included studies in this review were found “good quality” (scored >80% of the criterion of the NIH tool).

### 3.4. Baseline Characteristics of the Study Participants

The baseline characteristics of patients employed by the included articles are shown in Table 2. Among the total 8,320 study subjects, males were dominant (>50%). At enrollment, the mean age, weight, body temperature, and the hemoglobin level were ranges from 1.9 to 31.9 years, 8.7 to 40.3 kilograms, 38.1 to 39°C, and 8.7 to 12.5 g/dl, respectively. At enrollment, gametocytes were found in 2.1% to 13.9% of the study participants. Similarly, the average parasite count (the geometric parasite density, GMPD) per patient was between 7,898 and 65,299 in a microliter of blood. Parasite density (parasite/*μ*l of whole blood) was estimated using the following formula; “number of parasites counted”/“WBC counted” multiplied with “total WBC count/*μ*l” [32].

### 3.5. Treatment Outcome

**Figure 2:**
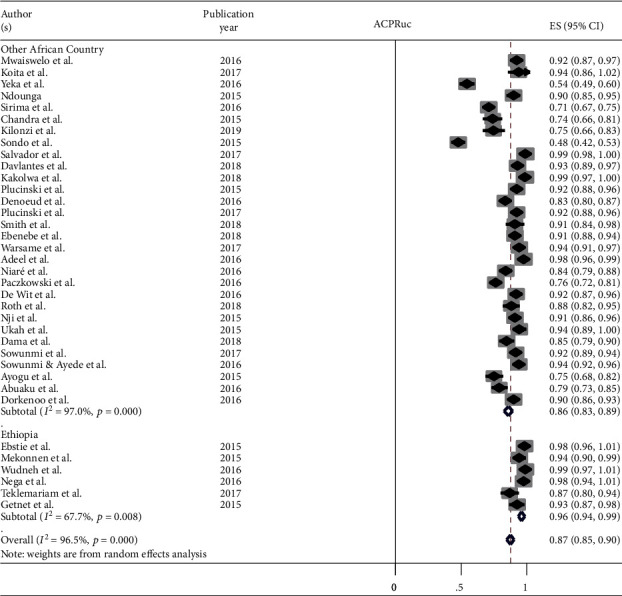
Forest plot for PCR-uncorrected ACPR, 2015-2019. ^∗^ACPRuc: Adequate Clinical and Parasitological Response _ PCR-uncorrected.

The overall treatment outcome is summarized in (Table 3). In the included studies, the primary endpoint while measuring the efficacy of AL was PCR-adjusted ACPR (or cure rate) on day 28. The secondary outcomes were fever and parasitemia clearance. Eight studies [33–40] did not report the PCR corrected ACPR. However, except two studies [41, 42], the rest reported PCR uncorrected ACPR and included for this group of analysis. Using meta-analysis, the PCR uncorrected ACPR of AL for the treatment of uncomplicated malaria was 87% (95% CI: 85-90%). Except a study by Sondo et al. that quantified 77.8% PCR corrected ACPR [28], the rest reported between 93.4 and 100% (Figure 2).

**Figure 3:**
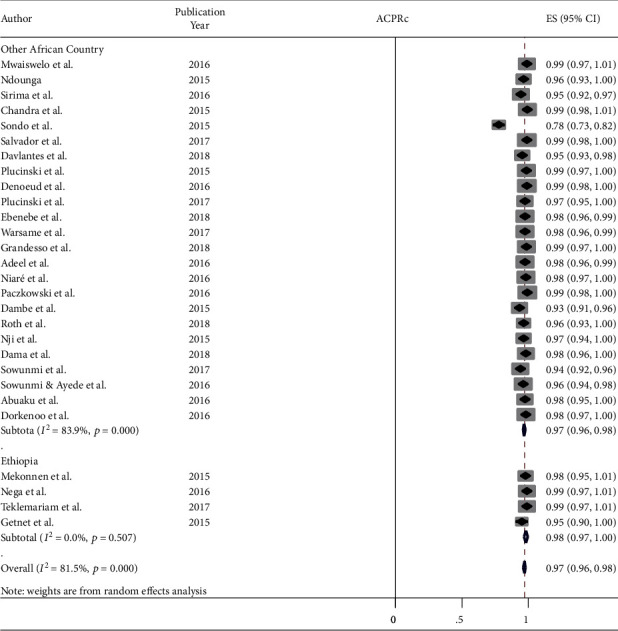
Forest plot for PCR-corrected ACPR, 2015-2019. ^∗^ACPRc: Adequate Clinical and Parasitological Response _ PCR-corrected.

Likewise, the pooled PCR corrected APCR was at 97.0% (95% CI: 96-98%) (Figure 3).

In this review, the overall early treatment failure (ETF) rate was almost 0%, while the proportion of late treatment failures (clinical and parasitological) was between 0% and 25.6% and 0% and 52.6%, respectively. The common type of treatment failure was late parasitological failure (52.6%) which was reported by only a study [10]. The reinfection rate within 28 days was ranged between 0% and 44.6%, and the recrudescence rate was between 0% and 6.1%. Reinfection is the development of malarial signs and symptoms due to a new strain, while recrudescence specifies the infection that has recurred from persistent blood stages of *P. falciparum*.

### 3.6. Fever and Parasite Clearance Rate

The distribution of fever and parasite clearance rates reported by some of the included articles is shown in Table 4. In this review, we noted that fever and parasite clearance was rapid. Almost all articles reported that patients were parasite and fever free at day three. On the third day of AL treatment, parasite clearance was >93% (of course most reported 100%) except a study that reported 80.4% which in fact reported 25.4% late parasitological failure [40]. Similarly, among 20 articles that reported the rate of fever clearance, about 13 reported 100% fever clearance on the third day. Except a study by Yaka et al. [34] that reported 88.6%, the rest reported >94% fever clearance at day 3. Yaka et al. reported 21.1% and 22.9% late clinical and parasitological failure too, respectively [34].

Finally, in this review, the most frequently reported adverse events associated with treatment with AL were cough, fatigue, weakness, anemia, GIT disorder (like abdominal pain, diarrhea, and vomiting), fever, and headache (Table 4).

## 4. Discussion

This review paper provides the latest data on the efficacy of AL (Coartem®), one of the ACTs for the treatment of uncomplicated falciparum malaria in African, where the efficacy of this drug has been less frequently evaluated especially in the last five years. More than 40 malaria-endemic countries in Africa were using ACT as first-line treatment for uncomplicated falciparum malaria [43].

Six dose regimen of AL over three days is the standard treatment for uncomplicated *P. falciparum* in most African countries [3, 44]. However, following the recently reported finding on the decreased falciparum parasite clearance with artemisinin derivatives in Southeast Asia (*Mekong Subregion*), specifically Cambodia and Thailand, there is a fear that the resistance may spread globally and it may pose a significant threat to malaria elimination. This emergence of artemisinin resistance has raised concerns that the most potent antimalarial drug may be under threat. Therefore, as per the WHO recommendation, there is a need for routine monitoring of the effectiveness of artemisinin derivatives in Africa for early intervention [14, 22, 45]. Consistent with the recommendation, the present review was conducted as part of the continued need of monitoring the therapeutic efficacy of AL in the treatment of uncomplicated falciparum malaria in the African context.

In this review, the primary efficacy endpoint was PCR-corrected ACPR (cure rate) at day 28. The pooled ACPR of AL for the treatment of uncomplicated falciparum malaria was at 87% (95% CI: 85-90%) before PCR adjustment and at 97.0% (95% CI: 96-98%) after adjustment. Except a study by Sondo et al. that reported 77.8% PCR corrected ACPR [28], the rest reported between 93.4 and 100%. According to the WHO recommendation, selection of an antimalarial drug as a drug of choice, it should have >/=90% parasitological and clinical cure rates [3]. Since adoption of AL as the drug of choice, many individual studies have been conducted in Africa [10, 13, 22, 27–31, 33–42, 44, 46–69] and Asia [14, 22] and reported high parasitological and clinical curatives capacity that fulfills WHO selection criteria. Our review is also in line with the WHO recommendation and a previous review article in Africa as well [70] which infers that the AL has maintained its efficacy since its introduction in the continent.

A global pooled analysis by Makanga et al. (2011) showed that the 28-day PCR-corrected parasitological cure rate (primary efficacy endpoint) was >97% for all age groups. This paper also reported that AL had rapid resolution of parasitemia and fever and also showed an excellent safety [71]. Another multicenter study in Asia and Africa reported >99% ACPR of AL in the treatment of uncomplicated falciparum malaria on day 28 [44]. These all findings confirm that AL is still an important drug that has been playing a major role as countries move towards the elimination of malaria. The ability of artemisinin to clear the biomass of *Plasmodium* within short hours of treatment and prevention of maturation of the gametocytes by the partner drug (lumefantrine) offers the maximum performance of AL [36]. Further advances in best practice of AL use would be considered through strategies to prolong the longevity of the drug and its improved access to people at risk of falciparum malaria [72].

In this review, the overall treatment failure rate was low; <10%. The early treatment failure (ETF) rate was almost 0%, while the proportion of late treatment failures (clinical and parasitological) was ranged between 0% and 52.6%. The common type of treatment failure was late parasitological failure (LPF) in which relatively higher proportion of such type of failure was reported by five studies; 12.3-25.4% [36, 40, 58, 59, 69] and one study at 52.6% [10]. The rest of the studies reported <8%. Treatment failure refers to the absence of resolution of parasitemia and clinical signs after antimalarial treatment, and true resistance to the drug [57]. It can be influenced by several factors more often a decrease in drug concentrations [42]. The reported low level of ETF and <8% late failures confirms the drug's efficacy and is emphasized by the rapid rate (within three days) of parasite clearance (>93%). AL clears parasites quickly as a result of the rapidly absorbed, fast-acting artemisinin component [39].

Day 3 parasitemia after treatment with a full dose of AL shown to be delayed parasite clearance [58] or a good indicator of sensitivity of *P. falciparum* to artemisinins [44]. The artemisinin component of AL is mostly responsible for the rapid parasite clearance [44]. Delayed parasite clearance is an early indicator of the emergence of resistance to artemisinin [31]. Data on parasite clearance is important to monitor the possible emergence of resistance to AL. In this review, fever and parasite clearance was quite rapid, notably within three days. Almost all articles reported that patients were parasite and fever free at day three. Except a study that reported 80.4% parasite clearance at day 3, which in fact reported 25.4% late parasitological failure [40], the overall parasite clearance in this review was >93%, which revealing a fast parasite clearance.

Among 20 articles that reported data on fever clearance, about 13 reported a 100% clearance rate on the third day. Except a study by Yaka et al. [34] that reported 88.6%, the rest reported >94% fever clearance at day 3. Yaka et al. also reported 21.1% and 22.9% late clinical and parasitological failure, respectively [34] that might explain its relative low level of fever clearance at day 3. Otherwise, the rapid fever clearance reported in our review could also be explained by the fast-acting parasite clearance properties of artemisinins, leading to rapid resolution of symptoms including fever, as explained previously. The fast fever resolving capacity of AL is also observed in other efficacy studies [71, 73]. In general, the high parasite and fever clearance rates reported in our review could be explained by the fast act of artemether to clear parasite biomass leading to rapid resolution of clinical manifestations [66].

In our review, during the 28-day follow-up, although AL almost cleared fever and parasitemia within 3 days, we have also noted some level of reinfection and recrudescence. Reinfection is the development of malarial signs and symptoms due to a new strain, while recrudescence specifies the infection that has recurred from persistent blood stages of *P. falciparum* [74]. Except four studies that reported 12.6-44.6% [34, 53, 58, 59] reinfection rate, most of the included papers reported <10%. Similarly, except two studies that reported 5.3% [60] and 6.1% [65] recrudescence rate within 28 days of follow-up, the rest reported <2.6% (the overall range was between 0% and 6.1%). From these figures, we can conclude that in this review most cases of late treatment failures were the result of reinfections as opposed to recrudescence; as our PCR corrected ACPR was >98%. This is of particular concern in areas with very intense malaria transmission where antimalarial drugs with longer half-life may offer the advantage of preventing reinfection but also be a target for the development of drug resistance [42]. This means that while treating malaria if there is parasite reappearance, it may be due to late clinical and/or late parasitological failures [55]. This result thus again confirms that the efficacy of AL for first-line treatment of uncomplicated falciparum malaria in Africa remains adequately high. However, the reported reinfection rate might suggest the continued need to scale-up effective malaria prevention interventions in Africa [59, 60].

The reported recrudescence rate in our review may reflect a decrease in the sensitivity of some *falciparum* strains to lumefantrine [39]. In the combination therapy of AL, artemether has a short half-life of about 1 hour. Lumefantrine has a half-life of 3–6 days and clears the long-lasting parasites and thus is expected to avoid the occurrence of recurrent parasitemia [3]. A recent study by Sowunmi et al. (2019) showed a declining parasitological response of AL through time. According to this study, by day 28, the risk of recurrent infections rose from 8 to 14% ten years following deployment of AL as first-line drug in Nigeria which may be due to emergence of parasites with reduced susceptibility or decrease in immunity to the infections among the study subjects [18]. While drug resistance can cause such a treatment failure, not all treatment failures are actually due to drug resistance. Factors like incorrect dosing, noncompliance with duration of dosing, poor drug quality, poor or erratic absorption, and misdiagnosis could contribute for failure [75]. However, our review did not put an attempt to figure out either of these factors as these may potentially contribute to the development and intensification of true drug resistance [75]. Therefore, in Africa where the AL is being used as first-line treatment, regular monitoring of its efficacy should be in place [39].

### 4.1. Strength and Limitations

To the best of our knowledge, this systematic review reported the latest therapeutic efficacy of AL for the treatment of uncomplicated malaria in African. However, the review should be interpreted in light of a couple of drawbacks; the absence of data from some African countries might compromise the overall picture of the current efficacy of AL in the continent. The other pitfall of this review is the heterogeneity of the articles in terms of the study design and the included age groups of the participants. Yet another notable limitation of the review is that it primarily considered the ACPR data of the 28 days of follow-up, the minimum period recommended by WHO for drugs with elimination half-lives of less than seven days [3]; any additional recurrences beyond this time frame were not considered. Data on the adherence and missing dose of AL was not included in the present review as these would upsurge the chance of recrudescence [59]. Finally, restricting our inclusion criteria that includes only articles published in the English languages may introduce missing relevant studies and reduced the precision of our results.

## 5. Conclusions

This review discovered that despite its introduction for more than a decade, AL is effective and thus could continue to be the drug of choice for the treatment of uncomplicated falciparum malaria in Africa for all age groups. This would imply no imminent threat of AL resistance development in the region. There may be a need to further investigate the comparatively low efficacy (<90%) rate reported by a study [28] in order to identify possible determinants of the reported treatment failure. However, there is no evidence at this time that a change in regimens is required. Therefore, concerned stakeholders should note that the threat of spreading from Asia or new development of resistance for AL warrants regular monitoring of its efficacy, possibly with plasma drug-level measurement, in order to detect any emerging new threat throughout the African region. On top of this, the reported reinfection rate in this review reflects the continued need to scale-up the effective malaria prevention interventions, such as the use of bed nets and other vector control measures.

## Figures and Tables

**Table 1:** Characteristic of the included studies, 2015-2019.

Author and year of publication	Country	Study design	Follow-up (in days)	Study subjects		
				Sample size	Male %	Included age groups
Mwaiswel, 2016	Tanzania	A randomized, single-blind clinical trial	28	110	50	>/=1 year
Koita, 2017	Mali	A randomized, clinical trial	42	33	100	≥18 years
Yeka, 2016	Uganda	A randomized trial	28	302	54	6 to 59 months
Ndounga, 2015	Congo	A randomized study	28	133	57.1	<10 years
Ebstie, 2015	Ethiopia	Observational cohort study	28	130	60	>5 years^∗∗^
Sirima, 2016	Sub-Saharan Africa	A randomized, multicentre, trial	63	407	49	<5 years
Chandra, 2015	Multi-center	A randomized, open-label study	42	131	50.4	6 to 59 months
Kilonzi, 2019	Tanzania	Prospective study	28	100	56	6 to 59 months
Sondo, 2015	Burkina Faso	A randomized open-label trial	28	340	53.8	All age
Salvador, 2017	Mozambique	Prospective one-arm study	28	349	52.3	Children
Davlantes, 2018	Angola	An in vivo assessment	28	185	54	Children
Kakolwa, 2018	Tanzania	Open-label single arm study	28	182	50	>/=6 months
Plucinski, 2015	Angola	Open-label, nonrandomized study	28	157	nr^∗^	6 months to 9 years
Denoeud,2016	Mali and Niger	Open comparative intervention study	28	397	47.8	< 5 years
Mekonnen, 2015	Ethiopia	Open-label single arm study	28	93	59.8	>6 months
Plucinski, 2017	Angola	Open-label single arm study	28	178	60	6 months to 12 years
Smith, 2018	Sierra- Leone	One-arm prospective study	28	64	65.6	6 to 59 months
Ebenebe, 2018	Nigeria	Open-label trial	28	324	53.7	<5 years
Warsame, 2017	Somalia	One-arm prospective study	28	284	66.9	6 months to 60 years
Grandesso,2018	Niger	One-arm prospective study	42	218	50	6 to 59 months
Adeel, 2016	Sudan	One-arm prospective study	28	595	48	All age group
Niaré, 2016	Mali	Randomized open-label assay	28	237	58.7	>/= 6 months
Paczkowski, 2016	Malawi,	Randomized efficacy trial	28	338	52.1	6 to 59 months
De Wit, 2016	D.R. Congo	Open-randomized trial	42	144	79	6 to 59 months
Dambe, 2015	Malawi	One-arm prospective study	28	322	49.6	6 to 59 months
Wudneh, 2016	Ethiopia	Open-label trial	28	91	82.4	>/=6 months
Roth, 2018	Kenya	A randomized controlled trial	28	96	50	6 months to ≤12 years
Nji, 2015	Cameroonian	Randomized trial	42	142	47.2	6 months to 10 years
Ukah, 2015	Nigeria	Double-blind randomized trial	28	75	0	Pregnant women
Dama, 2018	Mali	Randomized trial	42	158	53	All age group
Sowunmi, 2017	Nigeria	Randomized trial	42	517	57	</=15 years
Sowunmi, 2016	Nigeria	Randomized trial	28	517	55.1	</=15 years
Nega, 2016	Ethiopia	Open-label single-arm study	28	91	75.8	All age group
Teklemariam, 2017	Ethiopia	Open-label single-arm study	28	92	61.9	All age group
Ogouyèm2016	Benin	Open-label, single-arm trial	42	123	63	6 months to 5 years
Getnet, 2015	Ethiopia	One-arm prospective study	28	80	57.5	All age groups
Ayogu, 2015	Nigeria	A prospective study	28	154	22.7	All age group
Abuaku, 2016	Ghana	One-arm prospective study	28	170	55	6 months to 9 years
Dorkenoo, 2016	Togo	One-arm prospective study	28	261	54.8	6 to 59 months

^∗^nr: not reported, ^∗∗^yrs: years.

**Table 2:** Baseline characteristics of the study subjects with uncomplicated falciparum malaria in Africa, 2015-2019.

Author, year	Mean age in yrs	Mean wt in kg	Mean T in ^0^C	Mean Hgb in g/dl	GMPD	Presence of gametocytes (%)
Mwaiswel, 2016	10	34.3	38.3	—	8384	—
Koita, 2017	31.9	67.5	—	12.5	12000	—
Yeka, 2016	2.8	—	37.5	10.2	21616	13.9
Ndounga, 2015	5.4	19.6	—	10.5	30700	—
Ebstie, 2015	-NR	40.3	38.7	10.8	—	3
Sirima, 2016	2.5	—	38·7	9.5	65299.4	5
Chandra, 2015	2.7	12.8	—	—	—	—
Kilonzi, 2019	2.6	—	38.5	9.3	8745.8	—
Sondo, 2015	3.26	11	38.4	9	30529	—
Salvador, 2017	3.1	—	38.0	9.2	30 115	—
Davlantes, 2018	3	12	—	10.1	22340	—
Kakolwa, 2018	—	—	38.1	—	24400	—
Plucinski, 2015	—	—	—	—	—	—
Denoeud,2016	1.9	8.7	—	8.7	11200	—
Mekonnen, 2015	17.3	34.4	38.8	11.6	8404	2.2
Plucinski, 2017	6.4	18	—	10.2	20151	—
Smith, 2018	3.4	—	38.1	—	14 272	—
Ebenebe, 2018	3.3	13.4	37.9	10.1	16337	3.1
Warsame, 2017	12.3	—	38	—	9714	—
Grandesso,2018	2.5	10.5	38.9	9.7	46506	4.2
Adeel, 2016	—	—	38.2	—	11203	—
Niaré, 2016	9	22.7	—	10.9	—	—
Paczkowski, 2016	2.6	11.3	≥37.5	9.8	35512	—
De Wit, 2016	2.6	—	39.0	9.7	45154	—
Dambe, 2015	2.6	11.4	38.5	—	33080	2.1
Wudneh, 2016	13	41.5	37.9	13.7	13441.6	4.4
Roth, 2018	6.4	22.4	37.3	11.9	23672.5	4.17
Nji, 2015	4.8	17.8	38	9.9	14808	—
Ukah, 2015	29.4	—	—	—	12484	—
Dama, 2018	—	—	—	—	24325	—
Sowunmi, 2017	5	—	38.1	11.8	27 791	6
Sowunmi, 2016	4.5	—	38.1	9.8	24151.5	—
Nega, 2016	18.4	40.9	38.2	12.4	11509.6	5.5
Teklemariam, 2017	15.1	39.6	38.5	13.2	27798	7.6
Ogouyèm2016	2.6	—	38.7	8.9	42329	—
Getnet, 2015	19.4	35	38.3	12.3	7,898	10
Ayogu, 2015	—	—	—	—	49225	—
Abuaku, 2016	—	—	38.1	10.2	39,983	3.3
Dorkenoo, 2016	3	—	38.8	10	30498	4.5

yrs: years; wt: weight; kg: kilogram; ^0^C: degree Celsius; T: temperature; Hgb: hemoglobin; GMPD: Geometric mean parasite density per microliter of blood; nr: not reported.

**Table 3:** Summary of treatment outcomes of uncomplicated malaria treatment using AL in Africa, 2015-2019.

Treatment outcome		
Cure rate	ACPR PCRu	87% (95% CI: 85-90%)
	ACPR PCRc	97.0% (95% CI: 96-98%)

Treatment failure	ETF, %	0-2.5%
	LCF n, %	0-25.6%
	LPF n, %	0-52.6%

ACPR: adequate clinical and parasitological response; PCRu: polymerase chain reaction uncorrected; PCRc: PCR corrected; ETF: early treatment failure; LCF: late clinical failure; LFU: lost follow-up; LPF: late parasitological; n: final number; %: percent.

**Table 4:** Uncomplicated falciparum malaria treatment outcome including parasite and fever clearance rate, 2015-2019.

Author, year	% parasite clearance at day 3	% fever clearance at day 3	% treatment failure			% reinfection	% recrudescence	Most common AE
			ETF	LCF	LPF			
Mwaiswel, 2016	100	100	0	5.8	1.9	4.8	1.0	—
Koita, 2017	100	100	—			6	—	—
Yeka, 2016	100	88.6	0	21.1	22.9	44.6	2.5	Cough
Ndounga, 2015	—	97	0	4.2	5.9	6	3	Fatigue
Ebstie, 2015	96.1	87.9	0	0	1.6	—	—	Weakness
Sirima, 2016	94.6	94	0	25.6	52.6	—	—	Anemia
Chandra, 2015	>90		—	—	—	—	—	Abdominal pain
Kilonzi, 2019	100	100	0	1.3	22.7		—	—
Sondo, 2015	—	—	0.6	6.6	15	0.3	0.25	Cough
Salvador, 2017	99.1	—	0	1.2	0			—
Davlantes, 2018	100	—	0	8.6	—	3.8	1.5	—
Kakolwa, 2018	—	—	0	0	4.3		—	Cough
Plucinski, 2015	98.8	—	0.6	14.6	—	8.9	5.7	—
Denoeud,2016	—	—	0			15.2	1	GIT disorders
Mekonnen, 2015	100	100	0	1.1	4.5	2.2	2.2	—
Plucinski, 2017	100	—	1.7	15.7	—	3.4	0.6	—
Smith, 2018	100	—	—	0	8.9	—	—	—
Ebenebe, 2018	97.9	—	9			—	2.1	Cough
Warsame, 2017	100	—	0	0.7	0.3	—	—	Fever
Grandesso,2018	100	—	0	0.8	0.8	0.20	2.6	Fever
Adeel, 2016	100	—	1	0	0	—	—	—
Niaré, 2016	—	—	0	—	12.34	12.6	—	—
Paczkowski, 2016	99.7	—	0	23.8		23.1	0.7	—
De Wit, 2016	95.6	—	0	3.3	4.9	—	5.3	Asthenia
Dambe, 2015	99.1	—	1.6	4.1	0.9	—	0	URTI and pneumonia
Wudneh, 2016	100	100	0	0	1.2	0	1.2	Headache
Roth, 2018	97.5	100	0	0	3.6	0	1.2	
Nji, 2015	90	>90	0	0.8	2.4	—	—	Vomiting
Ukah, 2015	93.0	—	0	1.3	4	—	—	—
Dama, 2018	> 90.5	100	0	0	1.9	—	—	—
Sowunmi, 2017	100	100	0	6.7		—	—	—
Sowunmi, 2016	99.4	100	0	6.7		—	6.1	Cough
Nega, 2016	100	96.6	0	1.2	1.2	1.2	1.2	—
Teklemariam, 2017	100	100	0	1.3	0	—	—	Headache
Ogouyèm2016		100	0	8.9	4.1			—
Getnet, 2015	94.9	96.3	2.5	2.5	3.8	2.5	0	Headache
Ayogu, 2015	80.4	100	0	3.4	25.4	—	—	—
Abuaku, 2016	100	100	0	4.7	14.1	—	—	Diarrhea
Dorkenoo, 2016	100	—	0	0	1.5	—	—	—

ACPR: adequate clinical and parasitological response; PCR: polymerase chain reaction; ETF: early treatment failure; LCF: late clinical failure; LPF: late parasitological failure; AE: most frequent adverse event; URTI: upper respiratory tract infection.

## Data Availability

All generated data about the review are included in this manuscript. The original data can be accessed from the corresponding author at any time.

## Supplementary Materials

Supplementary Materials Supplementary 1 Search strategies used for the systematic review.
