# Supplementary material for: Therapeutic Efficacy of Artemether-Lumefantrine (Coartem®) for the Treatment of Uncomplicated Falciparum Malaria in Africa: A Systematic Review
Source: J Parasitol Res. 2020 Oct 20;2020:7371681. doi: 10.1155/2020/7371681 (PMC7599419; doi:10.1155/2020/7371681)
Supplement: Supplementary Materials — Supplementary 1 Search strategies used for the systematic review. [file 7371681.f1.docx]

**Supplement 1: Search strategy**

1. **PubMed; MeSH**

(((((("Therapeutics"[Mesh]) OR ( "Treatment Outcome"[Mesh] OR "Therapeutic Equivalency"[Mesh] )) AND "Artemether, Lumefantrine Drug Combination"[Mesh]) AND "Malaria, Falciparum"[Mesh]) AND ( "Africa"[Mesh] OR "Africa South of the Sahara"[Mesh] OR "South Africa"[Mesh] OR "Africa, Western"[Mesh] OR "Africa, Southern"[Mesh] OR "Africa, Northern"[Mesh] OR "Africa, Eastern"[Mesh] OR "Africa, Central"[Mesh] )))

1. **Advanced PubMed search with key words (41 articles)**

((((('Therapeutic efficacy') [Title/Abstract] AND 'artemether-lumefantrine')[Title/Abstract] OR Coartem)[Title/Abstract] AND 'uncomplicated falciparum malaria')[Title/Abstract] OR 'falciparum malaria'[Title/Abstract])

1. **SCOPUS**

( TITLE-ABS-KEY ( "artemether-lumefantrine" )  OR  TITLE-ABS-KEY ( coartem )  AND  TITLE-ABS-KEY ( "P.falciparum" )  OR  TITLE-ABS-KEY ( "Plasmodium falciparum" )  OR  TITLE-ABS-KEY ( "uncomplicated falciparum malaria" ) )  AND  ( LIMIT-TO ( AFFILCOUNTRY ,  "Kenya" )  OR  LIMIT-TO ( AFFILCOUNTRY ,  "Uganda" )  OR  LIMIT-TO ( AFFILCOUNTRY ,  "Nigeria" )  OR  LIMIT-TO ( AFFILCOUNTRY ,  "Burkina Faso" )  OR  LIMIT-TO ( AFFILCOUNTRY ,  "Mali" )  OR  LIMIT-TO ( AFFILCOUNTRY ,  "South Africa" )  OR  LIMIT-TO ( AFFILCOUNTRY ,  "Ethiopia" )  OR  LIMIT-TO ( AFFILCOUNTRY ,  "Senegal" )  OR  LIMIT-TO ( AFFILCOUNTRY ,  "Ghana" )  OR  LIMIT-TO ( AFFILCOUNTRY ,  "Mozambique" )  OR  LIMIT-TO ( AFFILCOUNTRY ,  "Zambia" )  OR  LIMIT-TO ( AFFILCOUNTRY ,  "Cameroon" )  OR  LIMIT-TO ( AFFILCOUNTRY ,  "Gambia" )  OR  LIMIT-TO ( AFFILCOUNTRY ,  "Congo" )  OR  LIMIT-TO ( AFFILCOUNTRY ,  "Cote d'Ivoire" )  OR  LIMIT-TO ( AFFILCOUNTRY ,  "Sudan" )  OR  LIMIT-TO ( AFFILCOUNTRY ,  "Benin" )  OR  LIMIT-TO ( AFFILCOUNTRY ,  "Gabon" )  OR  LIMIT-TO ( AFFILCOUNTRY ,  "Rwanda" )  OR  LIMIT-TO ( AFFILCOUNTRY ,  "Madagascar" )  OR  LIMIT-TO ( AFFILCOUNTRY ,  "Malawi" )  OR  LIMIT-TO ( AFFILCOUNTRY ,  "Egypt" )  OR  LIMIT-TO ( AFFILCOUNTRY ,  "Liberia" )  OR  LIMIT-TO ( AFFILCOUNTRY ,  "Togo" )  OR  LIMIT-TO ( AFFILCOUNTRY ,  "Niger" )  OR  LIMIT-TO ( AFFILCOUNTRY ,  "Sierra Leone" )  OR  LIMIT-TO ( AFFILCOUNTRY ,  "Democratic Republic Congo" )  OR  LIMIT-TO ( AFFILCOUNTRY ,  "Mauritania" )  OR  LIMIT-TO ( AFFILCOUNTRY ,  "Namibia" )  OR  LIMIT-TO ( AFFILCOUNTRY ,  "Zimbabwe" )  OR  LIMIT-TO ( AFFILCOUNTRY ,  "Burundi" )  OR  LIMIT-TO ( AFFILCOUNTRY ,  "Central African Republic" )  OR  LIMIT-TO ( AFFILCOUNTRY ,  "Eritrea" )  OR  LIMIT-TO ( AFFILCOUNTRY ,  "Somalia" ) )  AND  ( LIMIT-TO ( DOCTYPE ,  "ar" ) )  AND  ( LIMIT-TO ( LANGUAGE ,  "English" )
